# Diagnostic Utility of Specific Frailty Questionnaire: The Kihon Checklist for Hippocampal Atrophy in COPD

**DOI:** 10.3390/jcm13123589

**Published:** 2024-06-19

**Authors:** Tsunahiko Hirano, Shun Takahashi, Ayumi Fukatsu-Chikumoto, Kasumi Yasuda, Takuya Ishida, Tomohiro Donishi, Kazuyoshi Suga, Keiko Doi, Keiji Oishi, Shuichiro Ohata, Yoriyuki Murata, Yoshikazu Yamaji, Maki Asami-Noyama, Nobutaka Edakuni, Tomoyuki Kakugawa, Kazuto Matsunaga

**Affiliations:** 1 Department of Respiratory Medicine and Infectious Disease, Graduate School of Medicine, Yamaguchi University, 1-1-1 Minami-kogushi, Ube, Yamaguchi 755-8505, Japan; chiku05@yamaguchi-u.ac.jp (A.F.-C.); decem119@yamaguchi-u.ac.jp (K.D.); ohishk@yamaguchi-u.ac.jp (K.O.); j015ebponyou@gmail.com (S.O.); yomurata@yamaguchi-u.ac.jp (Y.M.); yyamaji@yamaguchi-u.ac.jp (Y.Y.); noyamama@yamaguchi-u.ac.jp (M.A.-N.); edakuni@yamaguchi-u.ac.jp (N.E.); kazmatsu@yamaguchi-u.ac.jp (K.M.); 2 Department of Psychiatry, Osaka University Graduate School of Medicine, Suita 565-0871, Japan; s.takahashi@psy.med.osaka-u.ac.jp; 3 Department of Neuropsychiatry, Wakayama Medical University, Wakayama 641-0012, Japanishidafine@gmail.com (T.I.); 4 Clinical Research and Education Center, Asakayama General Hospital, Sakai 590-0018, Japan; 5 Graduate School of Rehabilitation Science, Osaka Metropolitan University, Habikino 583-8555, Japan; 6 Department of System Neurophysiology, Wakayama Medical University, Wakayama 641-0012, Japan; tdonishi@wakayama-med.ac.jp; 7 Department of Radiology, St. Hill Hospital, Ube 755-0155, Japan; sugar@sthill-hp.or.jp; 8 Department of Pulmonology and Gerontology, Graduate School of Medicine, Yamaguchi University, Ube 755-8505, Japan; kakugawa@yamaguchi-u.ac.jp

**Keywords:** Kihon Checklist, hippocampal atrophy, diagnostic utility, COPD, daily inactivity

## Abstract

**Background/Objectives**: COPD patients who are frail have been reported to develop brain atrophy, but no non-invasive diagnostic tool has been developed to detect this condition. Our study aimed to explore the diagnostic utility of the Kihon Checklist (KCL), a frailty questionnaire, in assessing hippocampal volume loss in patients with COPD. **Methods**: We recruited 40 COPD patients and 20 healthy individuals using the KCL to assess frailty across seven structural domains. Hippocampal volumes were obtained from T1-weighted MRI images, and ROC analysis was performed to detect hippocampal atrophy. **Results**: Our results showed that patients with COPD had significantly greater atrophic left hippocampal volumes than healthy subjects (*p* < 0.05). The univariate correlation coefficient between the left hippocampal volume and KCL (1–20), which pertains to instrumental and social activities of daily living, was the largest (ρ = −0.54, *p* < 0.0005) among the KCL subdomains. Additionally, both KCL (1–25) and KCL (1–20) demonstrated useful diagnostic potential (93% specificity and 90% sensitivity, respectively) for identifying individuals in the lowest 25% of the left hippocampal volume (AUC = 0.82). **Conclusions**: Our study suggests that frailty questionnaires focusing on daily vulnerability, such as the KCL, can effectively detect hippocampal atrophy in COPD patients.

## 1. Introduction

Chronic obstructive pulmonary disease (COPD) is a leading cause of mortality and deteriorating health, often accompanied by various extrapulmonary manifestations such as physical inactivity, sarcopenia, nutritional disorders, and cardiovascular disease [1,2,3,4,5]. These comorbidities exacerbate the overall health burden of COPD [6,7,8,9]. Additionally, this clinical multisystem decline reflects an increased vulnerability to health deterioration known as frailty. The prevalence of frailty in patients with COPD is reported to be at 20–57% [10]. In particular, cognitive dysfunction is a significant concern, often occurring in conjunction with or because of systemic frailty in patients. In fact, our previous study showed that patients with COPD had a higher proportion of individuals with overlapping sedentary behaviors and cognitive impairment (motoric cognitive risk [MCR]) than those with asthma or healthy individuals [6]. This implies that the poor prognosis of COPD may be related to a connection between frailty and brain function.

The coexistence of cognitive dysfunction and frailty significantly increased the risk of severe health outcomes accompanied by a frailty cycle [11]. The hippocampus, which is crucial for cognitive function, may decrease in volume, leading to issues in memory formation and an overall decline in cognitive ability [12]. Hippocampal atrophy, commonly observed in Alzheimer’s disease and other dementias, is considered an indicator of cognitive decline [13,14]. We previously demonstrated through neuroimaging that systemic frailty in COPD patients is associated with brain atrophy, including reduced hippocampal volume, a condition referred to as ‘brain frailty’ [15].

However, given that neuroimaging is a specialized and challenging technique in a clinical setting, cognitive dysfunction and frailty in patients with COPD are often overlooked, leading to a vicious cycle. Therefore, early detection is crucial for breaking this cycle and underscoring the need for a simple and efficient screening tool to identify underlying pathophysiology.

The Kihon Checklist (KCL) is a comprehensive tool used to assess the health and functional status of elderly individuals. It was developed by the Japanese Ministry of Health, Labour and Welfare as part of a nationwide strategy to identify and address frailty in the aging population. The checklist includes a series of questions covering various domains such as physical function, nutrition, social engagement, and mental health. By evaluating these aspects, the KCL helps healthcare professionals identify individuals at risk of frailty and implement appropriate interventions to improve their overall well-being and quality of life. Its effectiveness has been validated in numerous studies, making it a valuable instrument in geriatric care and research. But to the best of our knowledge, no studies have been conducted to evaluate whether the KCL can be an effective tool for detecting hippocampal atrophy, and whether this is applicable in cases of COPD. Therefore, we hypothesized that the frailty scoring system would assist in identifying patients who should undergo brain imaging. This study aimed to explore whether a systemic frailty scoring system can contribute to the detection of brain frailty by focusing on hippocampal volume reduction in patients with COPD.

## 2. Materials and Methods

### 2.1. Study Subjects

The subjects included 20 healthy individuals and 40 patients with COPD recruited from the Yamaguchi Medical University Hospital. COPD patient data published in Biomedicine 2021 were used [15]. Ambulatory patients with COPD and healthy controls aged > 40 years at the Yamaguchi University Hospital were recruited for this cross-sectional prospective study. We did not perform power calculations to determine the optimal sample size for statistical significance because this was an exploratory study investigating the association between KCL and hippocampus volume in a small, enrolled sample. Patients with COPD were treated with long-acting bronchodilators following the guidelines of the Global Initiative for Chronic Obstructive Lung Disease (GOLD). COPD was defined according to a post bronchodilator forced expiratory volume in a 1 s/forced vital capacity (FEV1/FVC) ratio of <70% based on the GOLD guidelines. All patients were in a stable condition and had experienced no exacerbations for at least three months prior to the study. Patients with disorders that prevented them from completing the study assessments were excluded as follows: poor disease control; presence of other diseases that could affect walking, such as lower limb paralysis; requirement for long-term oxygen therapy; and presence of malignant tumors. This study adhered to the ethical standards of the Declaration of Helsinki and was approved by the ethics committee of Yamaguchi University (Institutional Review Board No. H28-031). It was registered at UMIN-ICDR (UMIN000024645). All participants received a thorough explanation of the study protocol and provided their written informed consent.

### 2.2. Evaluation of Frailty

Frailty was assessed using the KCL [16]. KCL is a self-reported comprehensive health checklist developed by a study group from the Japanese Ministry of Health, Labor and Welfare. It serves as a screening tool for frailty among individuals aged ≥ 75 years in the community [17] and among patients with COPD [18,19]. The KCL comprises 25 questionnaire items divided into seven subdomains (Table S1): KCL (1–5) focusing on daily living, KCL (6–10) on motor functions, KCL (11–12) on undernutrition, KCL (13–15) on oral functions, KCL (16–17) on social isolation, KCL (18–20) on dementia, and KCL (21–25) on depression. Frailty is defined as a total score of 8 or more, pre-frailty as a score of 4 or more, and a score of 3 or less is considered indicative of health [20].

### 2.3. Assessment of Hippocampal Volume

The hippocampal volume was assessed as previously described [15,21]. Structural magnetic resonance imaging (MRI) images were acquired using a 3.0T MR scanner (Achieva 3.0T Quasar Dual; Philips Medical Systems) equipped with an eight-channel SENSE head coil. A 3D fast field echo T1-weighted sequence was utilized, with parameters set at TR/TE = 7.0/3.3 ms, FOV = 256 mm, 200 slices, voxel size = 1.00 × 1.00 × 1.00 mm, and slice thickness = 1.0 mm. The hippocampal volume and estimated total intracranial volume (eTIV) in each hemisphere were obtained using FreeSurfer software (version 6.0, https://surfer.nmr.mgh.harvard.edu/ accessed 16 April 2023). The quality of the preprocessed images was visually inspected by S.T. and K.Y. Patients who fell into the lowest 25% in terms of hippocampal volume were defined as the bottom group.

### 2.4. Assessment of Pulmonary Function

According to the recommendations of the American Thoracic Society/European Respiratory Society, pulmonary function was assessed using a CHESTAC-8800 DN type (Chest Ltd., Tokyo, Japan) [22]. We used race-specific reference equations to calculate the % predicted pulmonary function. The severity of COPD was assessed using the GOLD classification [1].

### 2.5. Statistical Analysis

All statistical analyses were conducted using JMP Pro^®^ version 16.00 (SAS Institute Inc., Cary, NC, USA). For sample size calculation, a non-statistical method was adopted, setting the sample size based on the number of cases available at the participating facilities during the study period, with previous reports serving as a reference [15]. Continuous variables were presented as medians ± interquartile ranges, while categorical variables were presented as numbers and percentages, as appropriate. Comparisons between two continuous variables were performed using the Mann–Whitney U test. Comparisons between two categorical variables were conducted using Pearson’s chi-squared test. The correlation between the frailty score and hippocampal volume was examined using Spearman’s rank correlation coefficient. Spearman’s partial correlation was used to identify significant correlations among variables, controlling for eTIV, gender, age, and pack-years in healthy subjects as covariates. We conducted a likelihood ratio chi-squared test to assess the presence of significant differences across each distinct group. Sensitivity versus specificity was analyzed using the area under the curve (AUC), and a cut–off value for the KCL subdomain was determined to identify the bottom group. Statistical significance was set at *p* < 0.05 for all analyses.

## 3. Results

Baseline characteristics of the study participants are presented in Table 1. Among patients with COPD, there was a notably higher proportion of males, older individuals, and a greater smoking burden than in healthy subjects. Additionally, the COPD group exhibited more severe airflow limitation and frailty. According to the GOLD criteria, 36 of the 40 COPD patients (90%) were classified into mild and moderate stages. Moreover, approximately 80% of these patients exhibited frailty (pre-frail and frail), as assessed using the Kihon Checklist (KCL).

**Figure 1:**
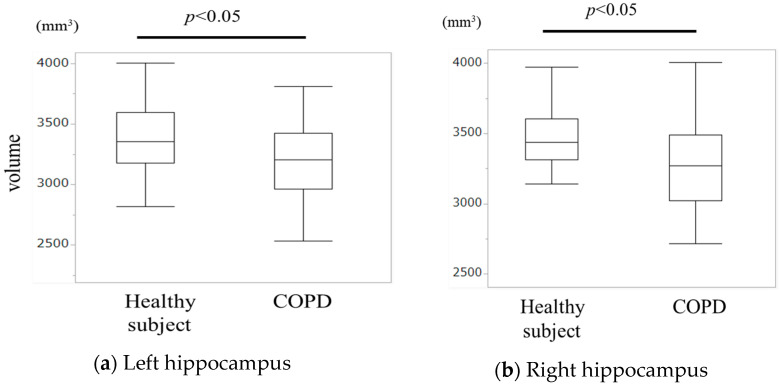
Comparison of hippocampal volume between healthy individuals and those with COPD. (**a**) Difference in the volume of the left hippocampus between healthy individuals and patients with COPD. (**b**) Difference in volume of the right hippocampus between groups.

The volumes of both the left and right hippocampi were significantly reduced in the COPD group compared with those in the healthy group (*p* < 0.05), as shown in Figure 1.

**Figure 2:**
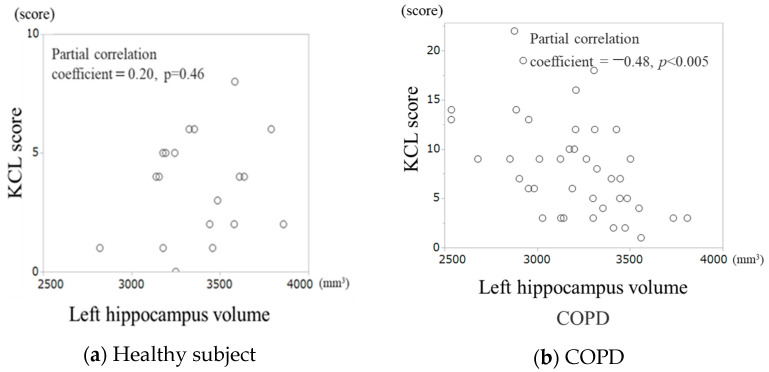
Comparison of correlation of frailty with left hippocampal volume in COPD. (**a**) Partial correlation analysis of KCL scores and left hippocampal volume in healthy individuals. (**b**) Partial correlation analysis of KCL scores and left hippocampal volume in COPD patients. Partial correlation analysis was adjusted for eTIV, gender, age, and pack-years in healthy subjects and adding GOLD stages for subjects with COPD as covariates.

Figure 2 presents the results of a partial correlation analysis adjusted for eTIV, age gender, and pack-years, showing a significant negative correlation between the left hippocampal volume and KCL scores in patients with COPD (Figure 2b, partial correlation coefficient = −0.48; *p* < 0.005), whereas no significant correlation was found in healthy individuals (Figure 2a). The right hippocampus showed a similar partial correlation (Table S2).

**Figure 3:**
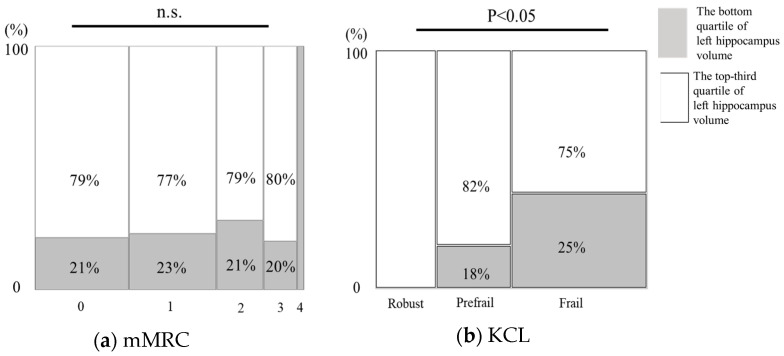
Discrimination ability of hippocampal volume reduction in COPD. (**a**) Correlation between left hippocampal volume and mMRC score. (**b**) Correlation between left hippocampal volume and KCL.

Figure 3 illustrates the distribution of hippocampal volume in the bottom and top third groups of patients with COPD stratified by mMRC (Figure 3a) and frailty by KCL (Figure 3b). Although no significant differences were observed in the distribution according to the degree of dyspnea, an increase in the distribution rates was noted with escalating levels of frailty severity (χ^2^ = 7.635, *p* < 0.05), with the following percentages: robust at 0%, pre-frail at 18%, and frail at 25%.

**Figure 4:**
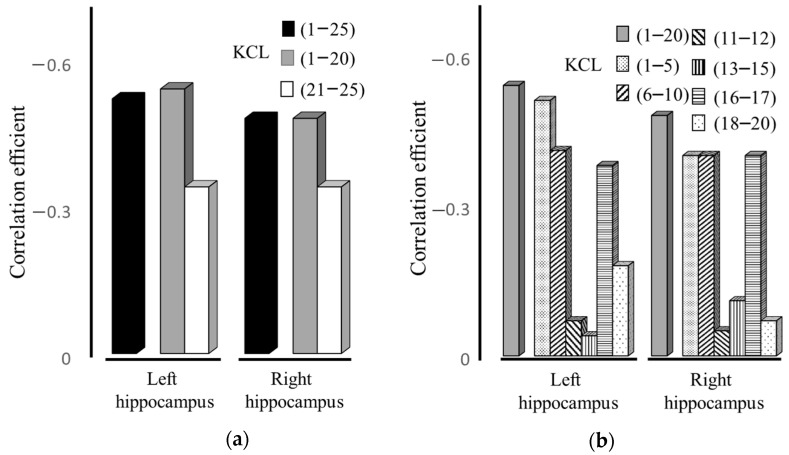
Correlation between hippocampal volume reduction and specific frailty-related behaviors in patients with COPD. (**a**) Correlation of left and right hippocampal volume with overall frailty domain [KCL (1–25)] and subdomain [(KCL (1–20), (21–25)]. (**b**) Correlation of left and right hippocampal volume with specific frailty subdomain [KCL (1–20), (1–5), (6–10), (11–12), (13–15), (16–17), 18–20)].

Table 2 and Figure 4 show the correlation between hippocampal volume and frailty scores across various subdomains. Notably, the scores from KCL (1–20) (left: ρ= −0.54, *p* < 0.0005, right: ρ = −0.48, *p* < 0.005) had a significantly greater impact on the overall KCL 1–25 scores than those from KCL (21–25) (left: ρ = −0.34, *p* < 0.05, right: ρ = −0.34, *p* < 0.05), particularly affecting the volume of the left hippocampus. Within the KCL (1–20) subset, scores for KCL (1–5), (6–10), and (16–17) were identified as the primary contributors.

**Figure 5:**
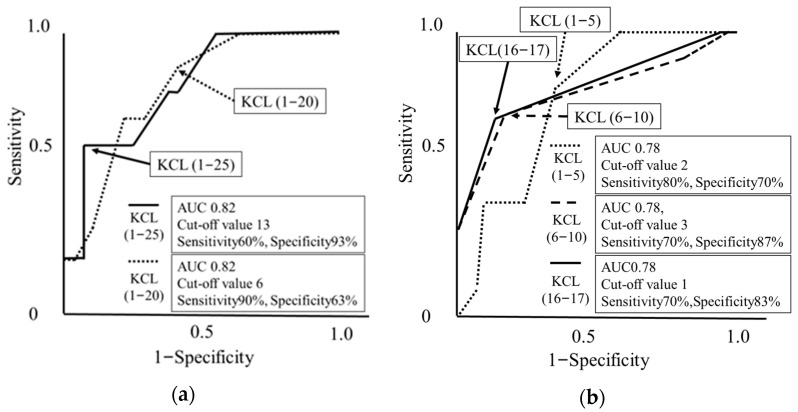
Utility of frailty total KCL and subdomains in capturing left hippocampal volume reduction in patients with COPD. This figure presents receiver operating characteristic (ROC) curves to evaluate the usefulness of overall frailty domain and subdomain scores [(**a**); KCL (1–25) and KCL (1–20), (**b**); KCL (1–5), KCL (6–10), and KCL (16–17)] in detecting left hippocampal volume reduction. In this analysis, the hippocampal volume reduction was defined as the bottom quartile.

As shown in Figure 5, the KCL subdomain scores of (1–25), (1–20), (1–5), (6–10), and (16–17) were validated as effective clinical instruments for identifying COPD patients whose left hippocampal volume falls in the lowest quartile. Each subdomain reached an area under the curve (AUC) of 0.78, indicating reliability. Specifically, achieving a score of 13 or higher or a score of 6 or higher in the KCL overall domain (1–25) and the KCL sub-domain (1–20), respectively, resulted in a specificity of 93% and a sensitivity of 90%. In the KCL subdomains (1–5) and (6–10), a score of 2 or higher or a score of 3 or more, respectively, resulted in a sensitivity of 80% and a specificity of 87%, and a score of 1 or higher in the KCL subdomain (16–17) attained 83% specificity in detecting individuals within the lowest quartile. Although the utility of total KCL frailty and its subdomains in capturing the trend of right hippocampal volume reduction in COPD is consistent, their ability to do so is slightly diminished (Table S3).

## 4. Discussion

In this study, we found a more pronounced correlation between frailty and hippocampal volume in COPD patients than in healthy individuals. Specifically, elevated frailty levels are associated with greater hippocampal atrophy. This correlation becomes especially evident when frailty assessments encompass evaluations of activities of daily living, motor function, and social isolation. Furthermore, our findings suggest that frailty questionnaires are efficient tools for detecting hippocampal vulnerability in COPD patients. To the best of our knowledge, this report is the first to demonstrate that KCL can be an effective tool for detecting hippocampal atrophy.

In our previous study, we demonstrated that frailty in individuals with COPD, along with a reduced quality of life, was associated with a decrease in hippocampal volume, as determined by MRI [15]. This observation aligns with the findings of the present study. Additionally, our research did not detect a similar pattern among healthy subjects and highlights the importance of focusing on comorbid conditions unique to patients with COPD. This discovery underscores a critical link between neurology and pulmonary medicine, revealing a relationship between the physical manifestations of COPD and changes in brain structure, specifically, a reduction in hippocampal volume. This suggests that the quality of life and frailty issues commonly observed in patients with COPD may stem from a neurological basis, as indicated by changes in the brain. These findings pave the way for a deeper understanding of the systemic effects of COPD, which extend beyond the respiratory system to affect the brain, potentially influencing cognitive function, emotional health, and overall neurological well-being.

The Kihon Checklist (KCL), which originated in Japan, is a frailty questionnaire recognized for its comprehensive approach to assessing frailty across physical, social, and mental dimensions [16]. Previous studies demonstrated a significant correlation between frailty and health status. This correlation was more pronounced than that with lung function, underscoring the complex relationship between frailty and QOL as opposed to respiratory function [18]. Our study revealed a specific association between hippocampal volume and vulnerabilities evaluated through the KCL, demonstrating that the correlation with items 1–20 (non-depression-related domains) was stronger than with items 21–25 (depression-related domains). This implies that within the context of COPD, a diminished hippocampal volume is more significantly associated with non-depressive vulnerabilities, including aspects of physical health, social connectivity, and cognitive function, rather than directly with depressive symptoms. Given the crucial role of the hippocampus in memory formation and emotional regulation, its link with non-depressive symptoms in COPD underscores the need to address a wide range of vulnerabilities in patients with this condition. Additionally, the study’s finding of a high prevalence of early-stage COPD suggests that these non-depressive neurological conditions in patients with COPD might manifest early in life, potentially leading to the subsequent development of depression as a secondary condition. COPD severity is positively correlated with the prevalence of psychological disorders, including depression and anxiety [23,24,25]. This suggests that, as COPD progresses, the likelihood of encountering mental health issues increases. In summary, the lower prevalence of mental disorders in the early stages of COPD than in the later stages might indicate that changes in neurological conditions that have not yet manifested as mental disorders begin in the early stages of COPD. This insight emphasizes the importance of early detection and holistic treatment approaches that consider the broad spectrum of neurological and respiratory health challenges in patients with COPD.

In this study, specific KCL subdomains that demonstrated the strongest correlation with hippocampal volume were identified through questions (1–5), (6–10), and (16–17). The first five questions, designated as KCL (1–5), pertain to instrumental activities of daily living (IADL), assessing the ability to execute daily tasks that demand both cognitive and physical capabilities. Questions (6–10) focus on physical strength and evaluate the individual’s muscular and physical endurance. Questions (16) and (17) aim to evaluate the seclusion domain, which addresses the aspects of social isolation [17]. The findings of this study highlight that lifestyle factors, as evaluated by items (1–5), (16–17), and physical strength, as assessed through items (6–10), are associated with decreases in hippocampal volume. This association highlights the vulnerabilities linked to these subdomains, suggesting that lifestyle and physical health are significant factors in the structural integrity of the hippocampus, especially in populations at risk of or diagnosed with conditions such as COPD.

The use of hippocampal atrophy rate as a metric is pivotal in differentiating individuals with mild cognitive impairment (MCI) who eventually develop Alzheimer’s disease (AD) from those who do not. Frankó and Joly [26] highlighted this significance, and Kasai [27] supported the notion that measuring the hippocampal volume is a critical and accessible method for diagnosing and monitoring the progression of various brain disorders. This is especially true as an early indicator of dementia before clinical symptoms manifest [26,28]. Despite the utility of hippocampal volume measurement, reliance on MRI technology presents significant drawbacks, including high cost and practical challenges in clinical settings. To address these limitations, the KCL test has been proposed as a cost-effective and brief screening tool. Unlike traditional screening methods, such as the Mini-Mental State Examination (MMSE) [29] and the Montreal Cognitive Assessment (MoCA-J) [30], the KCL test aims to identify patients who may require MRI for the detection of hippocampal atrophy, a condition that might not be evident through these conventional tools. This approach underscores the need for accessible and efficient methods for early identification and management of conditions leading to cognitive decline and dementia, facilitating timely and appropriate interventions. Moreover, the association between lifestyle and physical strength vulnerabilities and reduced hippocampal volume suggests that these vulnerabilities precede the onset of cognitive symptoms. This implies that interventions targeting lifestyle and physical strength may be effective in addressing cognitive decline in patients with COPD.

The disproportionate representation of males in the COPD group, likely reflecting the higher prevalence of COPD among males in Japan [31,32], comprises over 95%, compared to the healthy group, which consists of nearly 50% males. This raises concerns regarding the potential impact on the study results. The effect of gender differences on hippocampal volume and functional differences due to sleep disorders is noticeable [33,34]. As shown in Table S4, when the analysis was restricted to males, the hippocampal volumes on both sides tended to decrease in the COPD group compared to the healthy group (left: 3206 vs. 3441 mm^3^, right: 3250 vs. 3530 mm^3^); however, no significant differences were found (*p* = 0.28 and 0.26, respectively). This likely reflects the weak statistical power due to the small sample size. Therefore, future studies should include a larger number of female COPD patients as well as more healthy males. Furthermore, it is necessary to investigate the gender differences in the impact of hippocampal atrophy on QOL in COPD patients.

So far, it has been reported that frailty assessment using the KCL is useful in assessing frailty-related cerebral atrophy areas [35]. Moreover, KCL is shown to be associated with hippocampal volume reduction in cases of COPD [15]. However, to the best of our knowledge, no studies have been conducted to evaluate whether the KCL can be an effective tool for detecting hippocampal atrophy and whether this is applicable in cases of COPD. In this study, we found that KCL can be an effective tool for detecting hippocampal volume reduction, particularly in COPD cases. In the future, it is necessary to longitudinally examine whether KCL can predict the risk of future hippocampal atrophy. Additionally, investigating the causal relationships and detailed mechanisms behind these observations is essential. Furthermore, it is crucial to conduct intervention trials to determine if hippocampal atrophy can be reduced and whether KCL can accurately predict such reduction.

This study had several limitations. First, the small sample size limits the generalizability of our findings. Second, most of the study’s COPD patients belong to the GOLD I/II categories, so the effect of the frailty assessment was not evaluated in patients with severe COPD. Therefore, it is unclear whether the results of this study can be generalized to all COPD patients. Future studies need to include patients with varying degrees of COPD severity to confirm these findings. Third, owing to the cross-sectional nature of this study, the causal relationship between frailty and hippocampal reduction remains unclear. Fourth, no comparison has been made with other cognitive impairment questionnaires, such as the Hasegawa Dementia Scale and MMSE. Future research should aim for large-scale longitudinal studies to explore this connection more deeply. It would be beneficial to investigate the mechanisms driving this association, including the potential direct effects of COPD on neural health, shared risk factors, and indirect effects through systemic inflammation, oxygen deprivation, and other pathways. Additionally, examining whether interventions that improve COPD symptoms or overall physical health could counteract these brain changes might significantly enhance the patients’ quality of life and cognitive function. Finally, there was an age difference between the healthy and COPD groups, which may have impacted the results. However, even after adjusting for factors including age, the COPD group exhibited a significant correlation between KCL and hippocampal atrophy. This implies that, irrespective of actual age, there may be an inherent risk of structural brain abnormalities, potentially preceding the development of psychiatric disorders such as cognitive impairment at the time of COPD diagnosis. While aging is a well-known factor influencing most outcomes in our study, the significant relationship between frailty and hippocampal volume persisted even after adjusting for age. This finding suggests a robust association between the two variables. Furthermore, these findings underscore the potential of KCL, which is related to lifestyle, as a valuable screening and diagnostic biomarker for detecting hypothalamic atrophy. Ideally, matching the age of healthy subjects with those of COPD patients would be preferable, but the small sample size did not provide enough power to conduct a propensity-matching analysis. This limitation has been acknowledged above, and it is noted that this will be addressed in future studies.

## 5. Conclusions

Questionnaires specifically designed to assess frailty in a holistic manner, considering aspects such as daily lifestyle and physical, social, and psychological strengths, effectively identified hippocampal frailty in COPD patients. Furthermore, these aspects could be considered therapeutic targets, highlighting the necessity of a comprehensive management strategy for COPD that addresses the physical, social, psychological, and potential neuropsychological aspects of the condition.

## Figures and Tables

**Table 1:** Characteristics of the study subjects.

	Healthy Subject	COPD	*p*-Value
	(*n* = 20)	(*n* = 40)	
Gender (male/female)	7/13	39/1	<0.05
Age (years)	60 (57–72)	72 (64.5–76.8)	<0.005
BMI (kg/m^2^)	22.4 (20.4–24.3)	23.4 (20.4–25.0)	0.49
mMRC Dyspnea Scale (0/1/2/3/4)	12/8/0/0/0	14/13/7/5/1	<0.01
Smoking Status (Cu/Ex/Non)	1/5/14	12/28/0	<0.0001
Pack years	0 (0–20)	43.3 (27–58.9)	<0.0001
VC, %predicted	102.8 (96.6–115.8)	95.7 (85.5–113.9)	0.08
FVC, %predicted	107.4 (96.5–118.4)	97.3 (87.6–112.5)	0.07
FEV_1_, %predicted	105.8 (97.9–112.8)	75.1 (65.2–85.4)	<0.0001
DLco/VA, %predicted	92.3 (87.5–105.0)	75.1 (61.2–98.6)	0.07
GOLD(Ⅰ/Ⅱ/Ⅲ/Ⅳ)	-	15/21/4/0	
Kihon Checklist (KCL)	4 (1.3–5)	8.3 (±5.08)	<0.0005
Frailty using KCL	9/10/0	9/11/20	<0.001
(robust/prefrail/frail)			

Data are presented as the median (interquartile range) or n. BMI, body mass index; Cu, current smoker; Ex, ex-smoker; Non, non-smoker; VC, vital capacity; FVC, forced vital capacity; FEV_1_, forced expiratory volume in one second.

**Table 2:** Correlation with frailty and hippocampus in COPD.

			Left Hippocampus		Right Hippocampus	
	KCL		ρ	*p*-Value	ρ	*p*-Value
Overall domain	1–25		−0.52	<0.001	−0.48	<0.005
Sub- domain	1–20		−0.54	<0.0005	−0.48	<0.005
		1–5	−0.51	<0.001	−0.40	<0.05
		6–10	−0.41	<0.01	−0.40	<0.05
		11–12	−0.07	0.67	−0.05	0.78
		13–15	−0.04	0.80	−0.11	0.52
		16–17	−0.38	<0.05	−0.40	<0.05
		18–20	−0.18	0.27	−0.07	0.68
	21–25		−0.34	<0.05	−0.34	<0.05

## Data Availability

The data analyzed in this study are included in this article. Additional data are available from the corresponding authors upon request.

## Supplementary Materials

The following supporting information can be downloaded at: https://www.mdpi.com/article/10.3390/jcm13123589/s1, Table S1: Kihon Checklist (KCL); Table S2: Comparison of partial correlation analysis of frailty with right hippocampal volume between healthy subject and COPD; Table S3: Utility of frailty total KCL and subdomains in capturing right hippocampal volume reduction in COPD; Table S4: Comparison of hippocampal volume between healthy individuals and those with male COPD patients.
