# Supplementary material for: Diagnostic Utility of Specific Frailty Questionnaire: The Kihon Checklist for Hippocampal Atrophy in COPD
Source: J Clin Med. 2024 Jun 19;13(12):3589. doi: 10.3390/jcm13123589 (PMC11204603; doi:10.3390/jcm13123589)
Supplement: Supplementary file 1 [file jcm-13-03589-s001.zip › jcm-2973895-supplementary.pptx]

## Slide 1
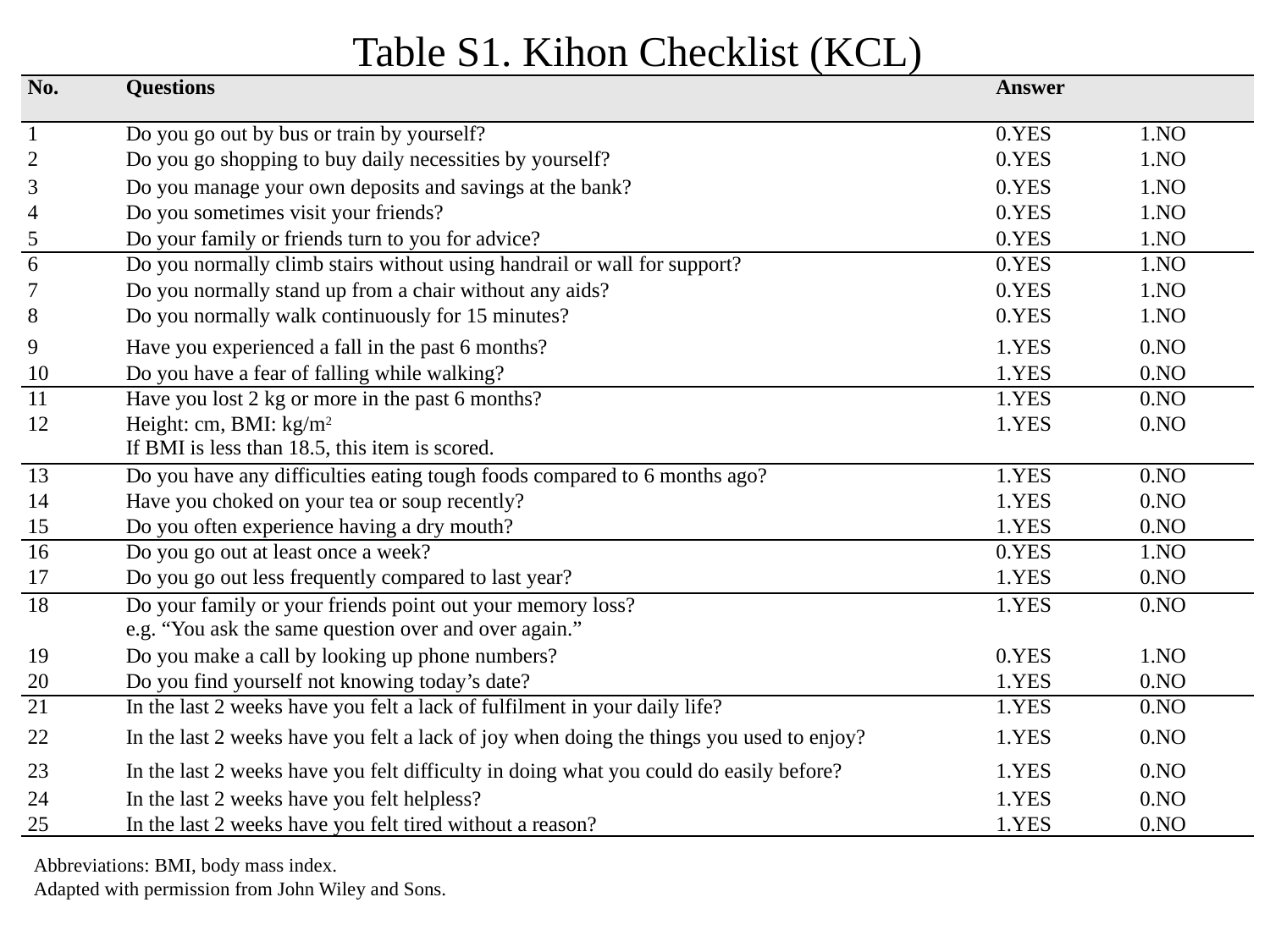

# Table S1. Kihon Checklist (KCL)
| No. | Questions | Answer | |
| --- | --- | --- | --- |
| 1 | Do you go out by bus or train by yourself? | 0.YES | 1.NO |
| 2 | Do you go shopping to buy daily necessities by yourself? | 0.YES | 1.NO |
| 3 | Do you manage your own deposits and savings at the bank? | 0.YES | 1.NO |
| 4 | Do you sometimes visit your friends? | 0.YES | 1.NO |
| 5 | Do your family or friends turn to you for advice? | 0.YES | 1.NO |
| 6 | Do you normally climb stairs without using handrail or wall for support? | 0.YES | 1.NO |
| 7 | Do you normally stand up from a chair without any aids? | 0.YES | 1.NO |
| 8 | Do you normally walk continuously for 15 minutes? | 0.YES | 1.NO |
| 9 | Have you experienced a fall in the past 6 months? | 1.YES | 0.NO |
| 10 | Do you have a fear of falling while walking? | 1.YES | 0.NO |
| 11 | Have you lost 2 kg or more in the past 6 months? | 1.YES | 0.NO |
| 12 | Height: cm, BMI: kg/m2 If BMI is less than 18.5, this item is scored. | 1.YES | 0.NO |
| 13 | Do you have any difficulties eating tough foods compared to 6 months ago? | 1.YES | 0.NO |
| 14 | Have you choked on your tea or soup recently? | 1.YES | 0.NO |
| 15 | Do you often experience having a dry mouth? | 1.YES | 0.NO |
| 16 | Do you go out at least once a week? | 0.YES | 1.NO |
| 17 | Do you go out less frequently compared to last year? | 1.YES | 0.NO |
| 18 | Do your family or your friends point out your memory loss? e.g. “You ask the same question over and over again.” | 1.YES | 0.NO |
| 19 | Do you make a call by looking up phone numbers? | 0.YES | 1.NO |
| 20 | Do you find yourself not knowing today’s date? | 1.YES | 0.NO |
| 21 | In the last 2 weeks have you felt a lack of fulfilment in your daily life? | 1.YES | 0.NO |
| 22 | In the last 2 weeks have you felt a lack of joy when doing the things you used to enjoy? | 1.YES | 0.NO |
| 23 | In the last 2 weeks have you felt difficulty in doing what you could do easily before? | 1.YES | 0.NO |
| 24 | In the last 2 weeks have you felt helpless? | 1.YES | 0.NO |
| 25 | In the last 2 weeks have you felt tired without a reason? | 1.YES | 0.NO |
Abbreviations: BMI, body mass index.
Adapted with permission from John Wiley and Sons.

## Slide 2
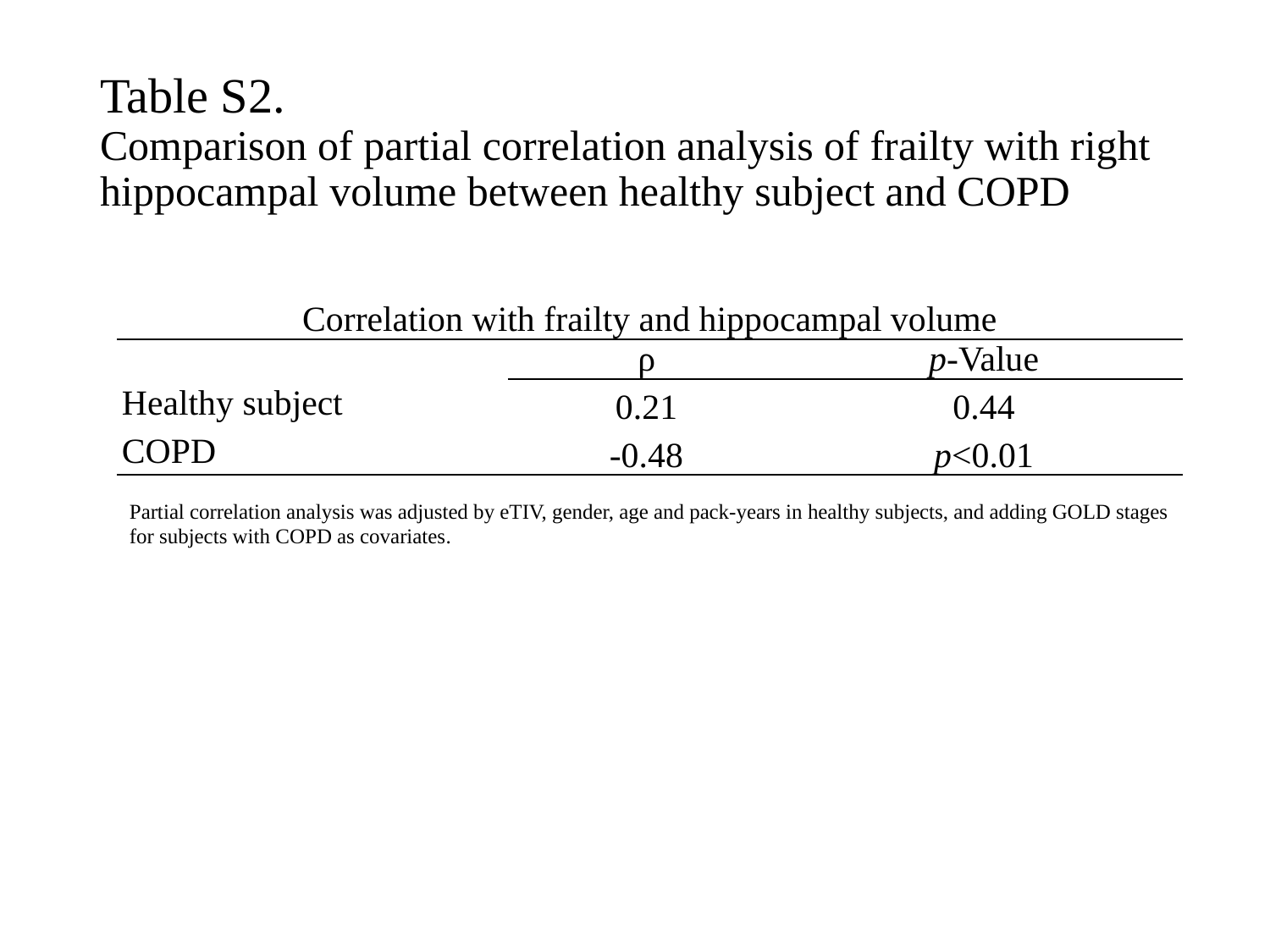

# Table S2. Comparison of partial correlation analysis of frailty with right hippocampal volume between healthy subject and COPD
| Correlation with frailty and hippocampal volume | | |
| --- | --- | --- |
| | ρ | p-Value |
| Healthy subject | 0.21 | 0.44 |
| COPD | -0.48 | p<0.01 |
Partial correlation analysis was adjusted by eTIV, gender, age and pack-years in healthy subjects, and adding GOLD stages for subjects with COPD as covariates.

## Slide 3
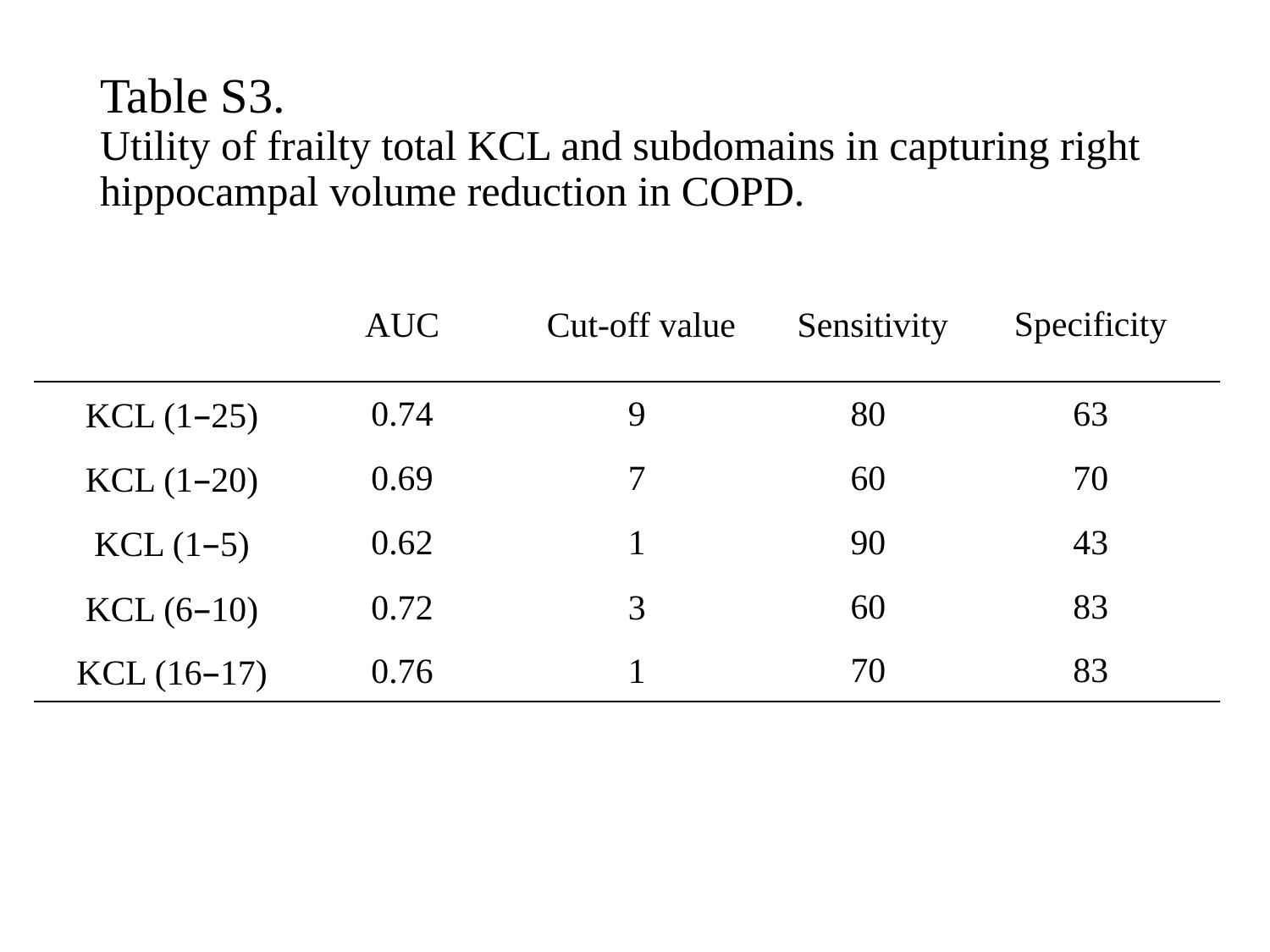

# Table S3. Utility of frailty total KCL and subdomains in capturing right hippocampal volume reduction in COPD.
| | AUC | Cut-off value | Sensitivity | Specificity |
| --- | --- | --- | --- | --- |
| KCL (1–25) | 0.74 | 9 | 80 | 63 |
| KCL (1–20) | 0.69 | 7 | 60 | 70 |
| KCL (1–5) | 0.62 | 1 | 90 | 43 |
| KCL (6–10) | 0.72 | 3 | 60 | 83 |
| KCL (16–17) | 0.76 | 1 | 70 | 83 |

## Slide 4
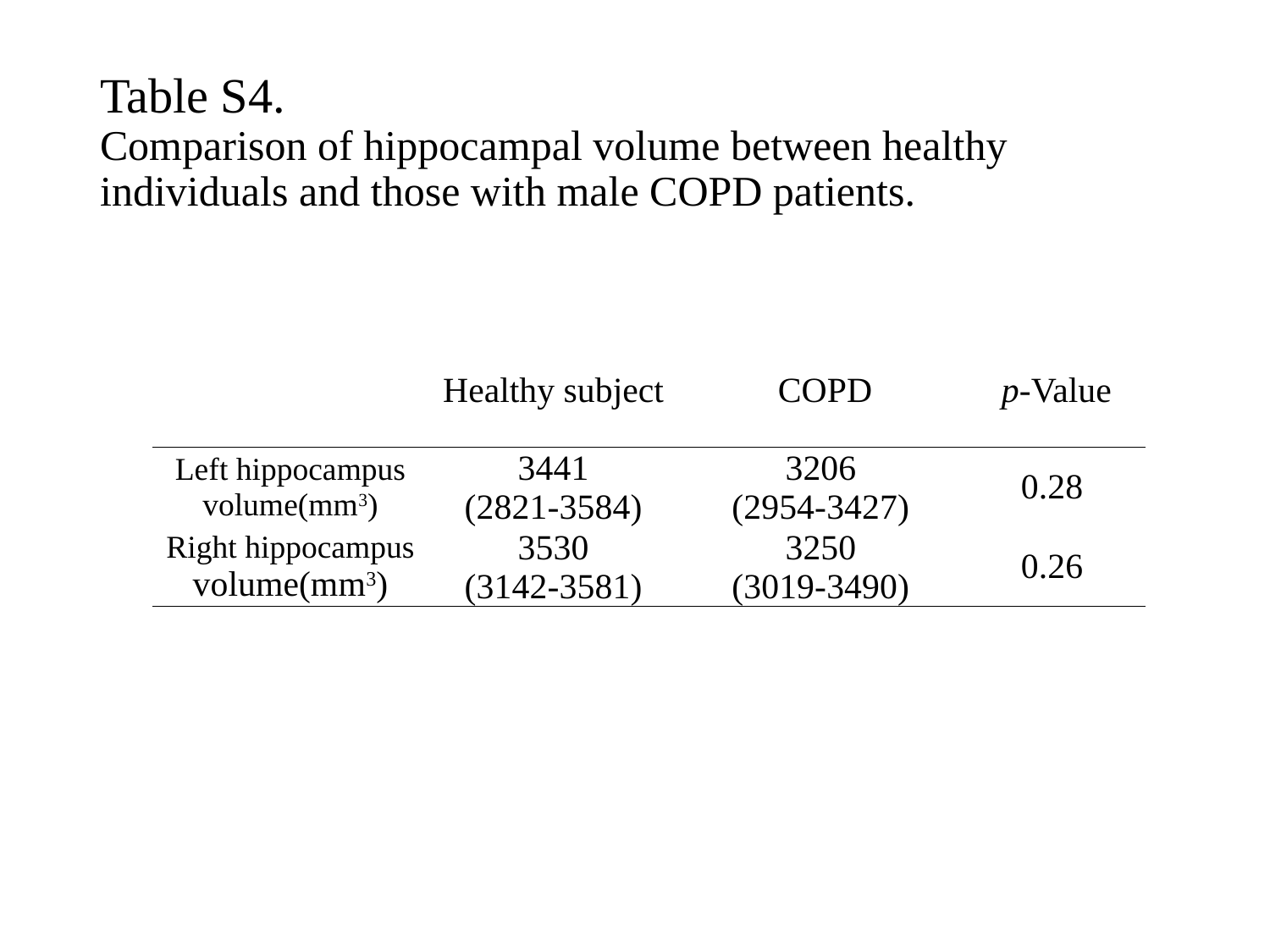

# Table S4. Comparison of hippocampal volume between healthy individuals and those with male COPD patients.
| | Healthy subject | COPD | p-Value |
| --- | --- | --- | --- |
| Left hippocampus volume(mm3) | 3441 (2821-3584) | 3206 (2954-3427) | 0.28 |
| Right hippocampus volume(mm3) | 3530 (3142-3581) | 3250 (3019-3490) | 0.26 |
